# Development and clinical validation of HPLC method for precise quantification of paclitaxel in plasma from breast cancer patients

**DOI:** 10.1371/journal.pone.0358891

**Published:** 2026-10-08

**Authors:** Najia Rahim, Sadia Shakeel, Maryum Nouman, Sadia Asim, Javeria Chaudhry, Zulqarnain Hassan

**Affiliations:** 1 Department of Pharmacy Practice, Dow College of Pharmacy, Dow University of Health Sciences, Karachi, Pakistan; 2 Department of Medical Oncology, Dow University Hospital, Karachi, Pakistan; 3 Dow Institute of Biological, Biochemical and Pharmaceutical Sciences, Dow University of Health Sciences, Karachi, Pakistan; Institute of Medical Sciences, Banaras Hindu University, INDIA

## Abstract

A simple, reproducible, and green high-performance liquid chromatography (HPLC) method was developed and validated for the precise quantification of paclitaxel in plasma following an intravenous dose in patients with breast cancer. The proposed method is developed to enhance efficiency, offering significant advantages including rapid analysis, one-step extraction, low solvent consumption, and thorough validation with extensive parameters. Chromatographic analysis was performed using Schimadzu HPLC with LC solution software. Plasma proteins were precipitated using acetonitrile for efficient extraction of paclitaxel. A separation was achieved on C18 column with the mobile phase being a combination of acetonitrile and water in the combination of 65:35 (v/v). The method exhibited excellent linearity (with a correlation coefficient of 0.9999) over the concentration range of 0.375 μg/mL to 8 μg/mL. The lower limit of quantification was found to be 0.375 μg/mL. The accuracy and precision of the intra-day (96.87–99.65%) and inter-day (98.3–99.9%) assays, comply with accepted bioanalytical criteria. The recovery of paclitaxel from plasma ranged between 96.97 and 100%. Stability studies indicated that paclitaxel remained stable after 24 hours at room temperature and 4 weeks at −20°C. AGREE, AGREEprep, ComplexGAPI, AMVI, AMGS, and ESA tools confirmed the environmental friendliness and greenness of the proposed method. Furthermore, the method was successfully utilized in the pharmacokinetic investigation of paclitaxel in breast cancer patients. The determined values of C_max_ were found comparable with reported values, confirming the method’s suitability for clinical pharmacokinetic study.

## Introduction

Breast cancer remains one of the most commonly diagnosed cancers globally. In 2020, 2.3 million women had a diagnosis of breast cancer, and 685,000 people died globally [1]. According to the American Cancer Society, taxanes are widely used in the management of breast cancer as both adjuvant and neoadjuvant chemotherapy [2].

Taxanes represent a class of chemotherapy drugs that exert their antitumor activity by obstructing the mechanism of cancer cell proliferation. These compounds are naturally derived from Yew trees. The taxane first introduced in clinical practice was isolated from the bark of the Western Yew tree and continues to play a major role in breast cancer treatment strategies [3].

The United States Food and Drug Administration (US-FDA) has approved two taxanes, paclitaxel (Taxol) and docetaxel (Taxotere) to treat breast cancer [4]. Although they share the same mechanism of action, the side effect profiles differentiate their clinical use. Paclitaxel is more commonly associated with peripheral neuropathy; however, docetaxel carries an increased risk of severe neutropenia and fluid retention. In addition, docetaxel often has a longer half-life in tumour cells. Although both paclitaxel and docetaxel are used to treat various malignancies, research indicates that their effectiveness may be equal [5]. However, the decision is frequently based on the particular type of cancer, the course of treatment, the patient’s pre-existing illnesses, and their tolerance to particular toxicities.

Paclitaxel (Fig 1) is a cytotoxic diterpenoid compound with an ester side chain at position C-13 and a taxane ring with a four-membered oxetane ring [6]. Its antineoplastic activity is mediated through binding to the β-subunit of tubulin, promoting tubulin polymerization and the formation of stable microtubules. By stabilizing microtubules against depolymerization, paclitaxel disrupts normal microtubule dynamics, thereby inhibiting interphase and mitotic cellular functions and ultimately inducing apoptosis [7,8]. Unlike other chemotherapeutic agents, paclitaxel is unique in that it attaches to microtubule polymers in cells in a particular, reversible, as well as saturable way [9]. Paclitaxel has proved clinical efficacy in a wide range of cancers, including ovarian, breast, head and neck, esophageal, Kaposi’s sarcoma, lung, bladder, prostate, cervical, and endometrial malignancies, and recommends an intravenous infusion of 100–175 mg/m^2^ over 1–3 hour infusion [10].

**Fig 1:**
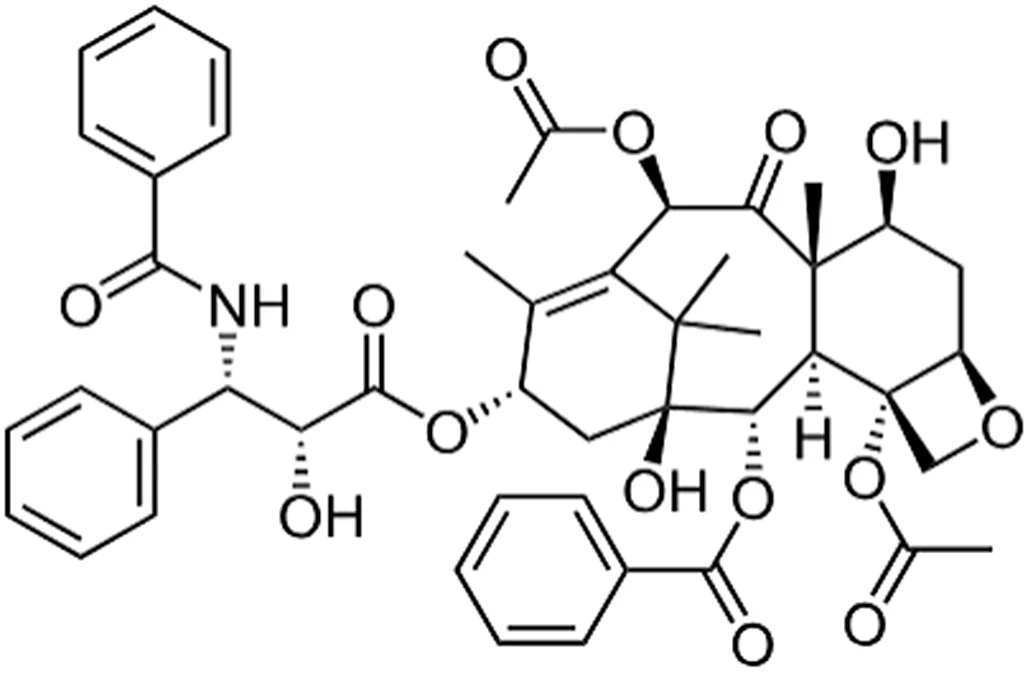
Structure of Paclitaxel.

The FDA approved paclitaxel in 1992, and due to its low aqueous solubility, it is formulated as non-aqueous concentrates, requiring dilution before being administered intravenously. The commercial formulation Taxol® consists of a 50:50 (v/v) mixture of dehydrated ethanol and Cremophor^®^EL (polyethoxylated castor oil).

Paclitaxel can be quantified in a biological matrix using various bioanalytical methods. Most reported methods rely on complex and expensive techniques like LCMS [11–14], which, although highly sensitive, may not be readily accessible in all laboratory settings. Researchers also utilized high performance liquid chromatographic (HPLC) methods for the quantification of paclitaxel in a biological matrix, however, several of these approaches involved the use of high organic solvent proportions, complex extraction methods, etc [15–19]. Accurate quantification of paclitaxel in different biological matrices requires a precise, simple, and accurate method, with an emphasis on reducing analytical time to increase accuracy and reliability.

The current method for determining paclitaxel from human plasma represents an advancement in routine bioanalytical practice. The proposed methodology is validated to improve efficiency compared to previously published methods, emphasizing rapid analysis, one-step extraction, and comprehensive validation with extensive parameters.

Furthermore, the method was applied to pharmacokinetic research in patients following intravenous administration of paclitaxel, to confirm its applicability in clinical research.

Green analytical chemistry emphasizes eco-friendly analytical methods, emphasizing reducing hazardous chemicals, minimizing waste, and conserving energy [20]. The sustainability of the proposed HPLC method was judged using the greenness metric tools, providing an objective evaluation of its environmental impact.

## Experimental

### Material and instruments

Fujian South Pharmaceutical Co. Ltd, China, generously provided a pure standard of paclitaxel. Acetonitrile (HPLC grade), trichloric acetic acid, and methanol were acquired from Merck in Darmstadt, Germany. The chromatographic analysis was performed with a Schimadzu HPLC 20A with LC solution software with a Merck C18 (250 mm × 4.6 mm) column. A microcentrifuge (DLAB D1524R-, China), vortex (Topsien Mivot, China), and analytical balance (PANTHER-USA) were also employed.

### Standard solutions

A primary stock solution was prepared by dissolving pure paclitaxel (10 mg) in 10 ml of methanol to obtain a concentration of 1 mg/ml. This solution was further diluted to get the concentration of 100 µg/ml (working solution). Serial dilutions were prepared from the working solution as required for the calibration curve and quality control (QC) samples.

### Plasma sample

Paclitaxel-spiked plasma samples were prepared by taking plasma (500 µl) in Eppendrof tubes and mixing it with the appropriate volume of paclitaxel solutions using a vortex for 2 min. Protein extraction was carried out by adding 500 µl of acetonitrile, followed by centrifuging at 15000 rpm for the duration of 15 min. The supernatant was then collected for HPLC analysis.

### Chromatographic conditions

Paclitaxel was analyzed using a C18 (5 µm, 250 mm × 4.6 mm) column at the detection wavelength of 227 nm. The mobile phase was comprised of acetonitrile and water in the ratio of 65:35 (v:v). The flow rate was maintained at 1 ml/min with the injection volume of 30 µl. Column temperature and total run time were 40°C and 10 min.

### Method validation

The proposed method was validated according to FDA and ICH M10 guidelines [21,22]. Validation parameters included sensitivity, selectivity, linearity, recovery, precision, accuracy, and stability.

### Calibration curve and linearity

Linearity was assessed by preparing calibration standards in blank plasma across the intended quantitation range. The peak response was plotted against the paclitaxel concentration, and regression analysis was performed (the acceptable limit is R^2^ > 0.9). Calibration curves (n = 3) were constructed to confirm the linearity. Percent accuracy, mean, SD, and %COV were calculated from the back-calculated concentrations.

### Selectivity and specificity

Paclitaxel-spiked plasma and blank plasma were analyzed and chromatograms were compared to confirm the selectivity and specificity of the method.

### Sensitivity, LOD and LOQ

Serial dilutions of paclitaxel in plasma (8–0.375 µg/ml) were analyzed to determine the limit of detection (LOD) and lower limit of quantification (LLOQ).

### Recovery

The extraction recovery was calculated by comparing the peak area plasma quality control (QC) samples with those of equivalent concentrations prepared in methanol, accounting for dilution factors. Recovery was evaluated for three independent days.

### Accuracy and precision

Accuracy represents the closeness of measured value to the known true value under prescribed conditions; however, precision is the closeness of agreement among a series of measurements acquired from multiple samplings of the same sample under specified conditions. Each calibrator was analyzed five times, and each QC sample was analyzed in triplicate to assess accuracy and precision.

### Stability studies

Stability studies were conducted to evaluate that paclitaxel extracted from human plasma samples remains stable at the different handling and storage conditions, with acceptable precision (COV%) and accuracy (%).

### Stock solution stability

To assess stock solution stability, stock solution (100 μg/ml) of paclitaxel was prepared and stored for 4 weeks at −20 ± 3 °C.

### Auto sampler stability

The auto-sampler stability helps in determining the stability of the active analyte within the auto-sampler. QCH and QCL samples were placed in the auto-sampler tray and analyzed at 0 hr, 3 hrs, 6 hrs, 10 hrs and 24 hrs.

### Bench-top stability

Bench-top stability determined the stability of the active analyte, i.e., at room temperature. Extracted QCH, QCM and QCL samples were kept at room temperature and analyzed at 0 hr, 3 hrs, 6 hrs, 10 hrs and 24 hrs.

### Freeze and thaw stability

Freeze-thaw stability was evaluated to determine the effect of multiple freezing and thawing cycles on analytic activity prepared in the plasma matrix. QC samples were prepared and initially analyzed at 0 hour and then stored at -20 ±5°C. After 24 hours of the initial analyses, the stored QC samples were taken out of the freezer and allowed to thaw unassisted at room temperature, followed by reanalysis. This procedure was repeated for additional freez-thaw cycles, and samples were analyzed at 24 hrs, 48 hrs, 72 hrs, and 96 hrs after the initial analysis to assess stability.

### Long term stability

Long-term stability determines the stability of the analyte, i.e., plasma matrix, upon storage (-20 ±5°C) for a long period of time (one month or more) depending upon the nature and behaviour of the drug. QC samples (QCH, QCM & QCL) in plasma were prepared and stored at -20 ±5°C, Analyst took out a sample every week, extracted it according to the procedure, and analyzed it to assess long-term stability.

### Matrix effect

Matrix effect is the change in the analyte response due to interference with unidentified components in the sample matrix. QCH and QCL samples were prepared using plasma from six different sources and analyzed to observe interfering matrix components.

### Dilution integrity

It is an assessment of the sample dilution procedure to affirm that it does not impact accuracy and precision. Samples of paclitaxel’s concentrations with 2 dilution factors (16, 8, 4 μg/ml) were prepared and analyzed.

### Carry-over effect

Carry-over was evaluated by analyzing QC samples prepared in plasma, followed by an injection of drug-free plasma, and the chromatograms were analyzed for interference peaks at the paclitaxel retention time.

### Application to breast cancer patients

In a clinical study approved by the Institutional Review Board (IRB) of Dow University of Health Sciences, Pakistan (Reference: IRB-4026/DUHS/Approval/2025/307, dated 12^th^ August 2025), the proposed HPLC method was used to assess the pharmacokinetic of paclitaxel in breast cancer patients. Female patients of age 18–75 years, diagnosed with breast cancer and prescribed with paclitaxel for chemotherapy were recruited from November’ 2025 to December’ 2025. Patients with pre-existing peripheral neuropathy, simultaneous anticancer treatment with hormone or immunotherapy, and severe hepatic/renal impairment were excluded.

Participants were briefed about the rational of the study and study procedure and written informed consent was obtained prior to enrolment. After the informed consent, a trained staff nurse will collect the blood sample (3 ml) in per-labled EDTA-containing anticoagulant tubes. Blood samples were collected at pre-dose and at the end of paclitaxel intravenous infusion. After centrifugation at 4000 g for 5 minutes, the plasma was separated and stored for analysis at −20°C. C_min_ and C_max_ were determined and compared with reported values.

## Results

### Optimization of chromatographic conditions and extraction methods

The HPLC method was developed and optimized by evaluating different chromatographic settings. Initially, different combinations of water, acetonitrile, and methanol were tested. Optimal peak shape and resolution were achieved with acetonitrile:water (65:35) as the mobile phase with the flow rate of 1 ml/min at a detection wavelength of 227 nm. For pharmacokinetic (PK) investigations, the plasma sample volume was minimized to 0.5 ml. Protein precipitation using 6% trichloric acetic acid caused turbidity; therefore, acetonitrile was selected as the extraction solvent based on the highest recovery findings (500 μl plasma sample: 500 μl acetonitrile).

### Method validation

#### Selectivity.

Six blank plasma samples were processed using the described extraction technique. There is no peak observed at the retention time of paclitaxel, demonstrating the method’s selectivity (Fig 2).

**Fig 2:**
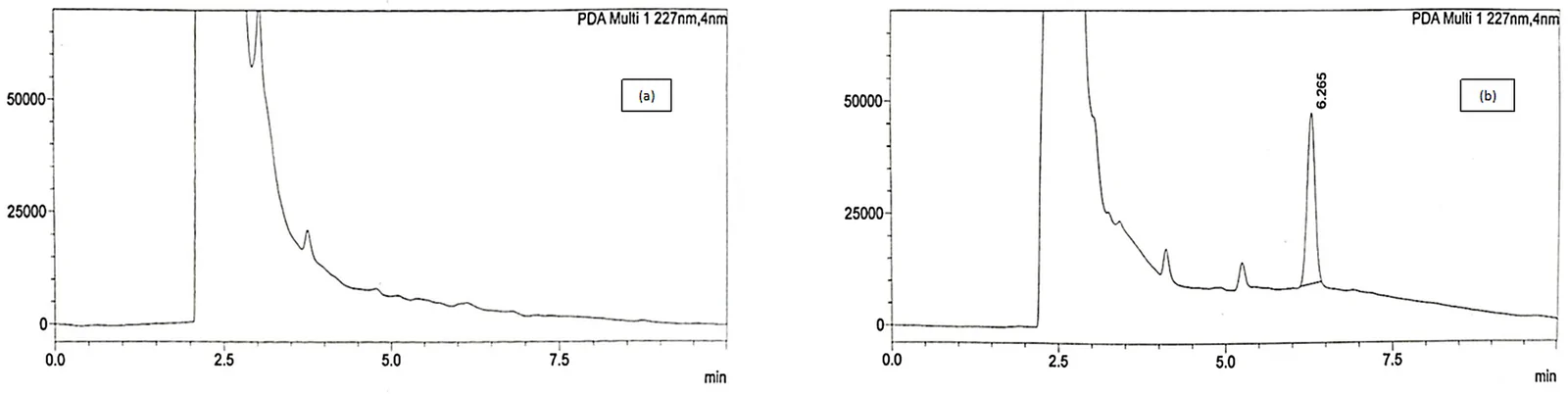
HPLC chromatograms of blank plasma (a) and paclitaxel spiked plasma (b).

The calibration curve was constructed in triplicate using the serial dilution (concentration range: 0.375–8 µg/ml), exhibiting excellent linearity (R^2^ = 0.9999; Fig 3 and Supp. Table 1 in S1 File). The lowest limit of quantification (LLOQ) for paclitaxel that provided adequate accuracy and precision was 0.375 µg/ml (Supp. Table 2 and Supp. Figure 1 in S1 File).

**Fig 3:**
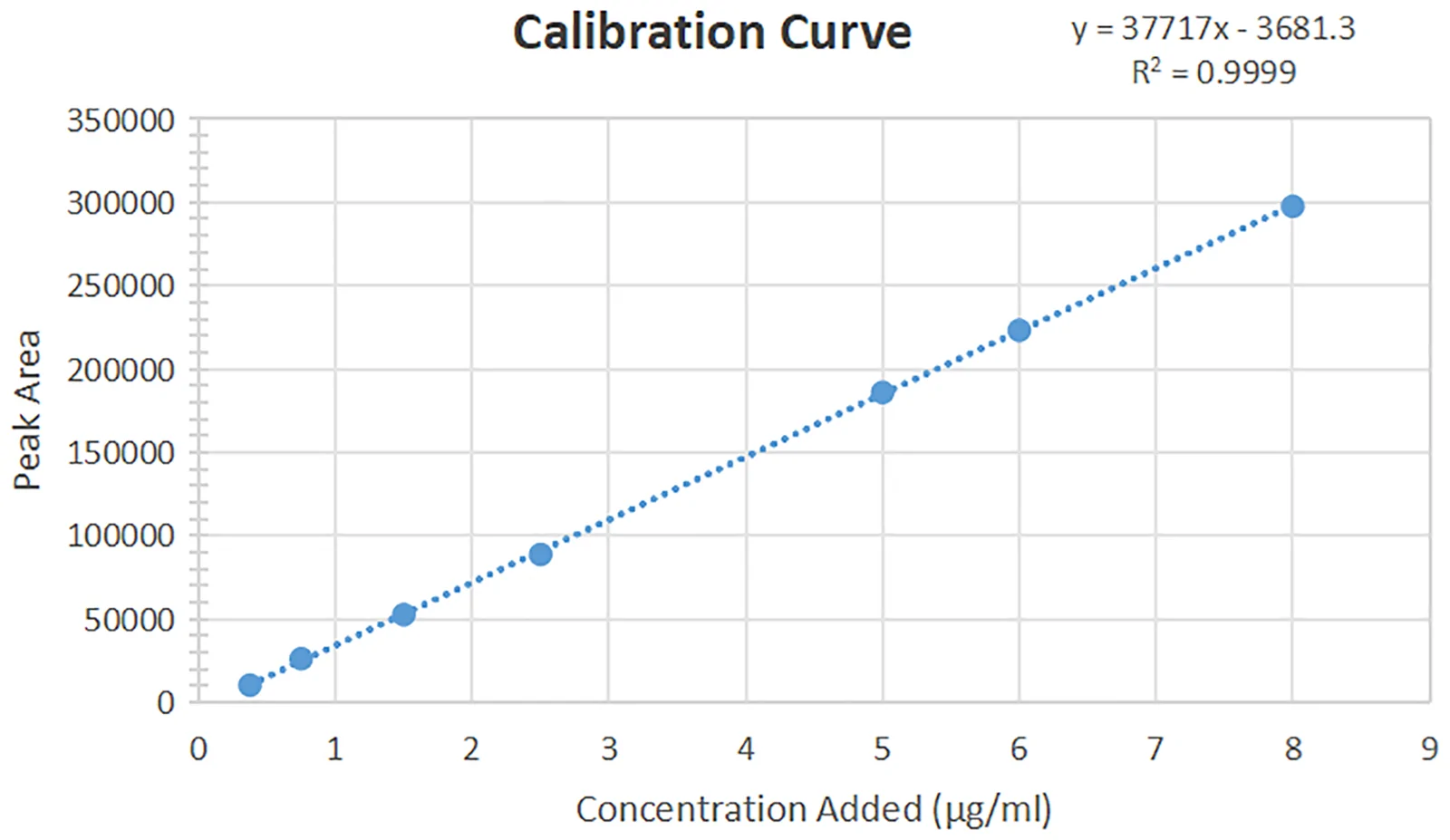
Calibration curve with linear equation.

The accuracy of LLOQ (0.375ug/ml) ranged from 94.37% to 104%, which is within acceptable limits (i.e., ± 20%) (Supp. Table 2 in S1 File). Sensitivity assessment disclosed that the method is able to quantify paclitaxel at the LLOQ of 0.375 ug/ml with acceptable accuracy and precision (i.e., ± 20%).

#### Recovery.

Quality control (QC) samples at 7, 4, and 1 µg/ml were analyzed, yielding the average recoveries of 96.97%−100.00% (Table 1). These results indicated that the analytical method is reliable and capable of providing accurate recovery for quality control across multiple days.

**Table 1:** Percentage recovery of paclitaxel from biological matrix (plasma).

Conc. added (µg/ml)		Mean peak area in solvent	Mean peak area in matrix	Dilution Factor	Recovery %	Mean±SD of recovery %	% COV
QCH*	7	460118	229107	2	99.59	99.71 ± 0.234	0.234
		483556.67	240720		99.56		
		470749.33	235326.67		99.98		
**QCM**	4	237661.33	117647.67		99	99.4 ± 0.525	0.528
		244543	122277.33		100		
		263930.67	130930.33		99.22		
**QCL**	1	53342	25862.33		96.97	98.75 ± 1.54	1.55
		53663.33	26705.33		99.53		
		53237	26551.33		99.75		

*QCH = quality control high, QCM = quality control medium, QCL = quality control low.

#### Accuracy and precision.

The proposed method demonstrated excellent accuracy and precision, with the percentage of accuracy and coefficient of variation (%COV) as 96.87–99.65% and 1.94–2.16 for intra-day and 98.3–99.9% and 0.34–1.63 for inter-day, respectively (Table 2). All results were within the FDA and ICH limits [21,22].

**Table 2:** Accuracy and precision of the HPLC method.

	Quality control samples							
	Intra-day	Inter-day	Intra-day	Inter-day	Intra-day	Inter-day	Intra-day	Inter-day
**Conc. added** **(µg/ml)**	8		7		6		5	
**Conc. Calculated (mean±SD)**	7.97 ± 0.16	7.87 ± 0.27	6.78 ± 0.14	6.92 ± 0.14	5.87 ± 0.117	5.9 ± 0.19	4.85 ± 0.09	4.95 ± 0.04
**%Accuracy**	99.65	98.3	96.87	98.81	97.91	98.4	97.08	99.9
**%COV**	2.00	1.63	1.94	0.34	1.99	1.27	1.94	0.82

#### Stability studies.

The paclitaxel stock solution (100 μg/mL) remained stable for up to four weeks at −20 ± 3 ^0^C with measured concentrations of 97.70%, 104.14%, 100.94%, and 105.15% at weeks 1, 2, 3, and 4, respectively (Supp Table 3 in S1 File).

Auto-sampler stability of QCH, QCM, and QCL samples over 24 hrs showed an accuracy of 96.31%, 97.23%, and 96.02%, respectively, confirming stability under these conditions (Supp Table 4 in S1 File).

Bench-top stability confirmed the accuracy of 97.31% bench-top stability of the QCH sample at 24 hr. The accuracy of bench-top stability of QCM and QCL samples at 24 hrs was 98.03% and 91.07% (Supp Table 5 in S1 File). These results show that the samples remained stable over 24 hrs on a working bench, indicating suitability for routine handling.

Freeze-thaw stability over four freeze- and -thaw cycles exhibited the accuracy of 105.07% (QCH), 109.69% (QCM), and 109.53% (QCL) (Table 3), confirming the sample integrity during recurrent freeze-thaw cycles.

**Table 3:** Freeze-thaw stability and long-term stability at −20°C.

Freeze-thaw Stability										
Conc. added (µg/ml)		Mean peak area		% Accuracy	Mean peak area	% Accuracy	Mean peak area	% Accuracy	Mean peak area	% Accuracy
		0 hour	24 hours		48 hours		72 hours		96 hours	
QCH*	7	186875.3	181920.7	97.35	190988.7	102.20	198681	106.32	196349	105.07
**QCM**	4	103337	99892.7	96.67	98812	95.62	112277	108.65	113346.7	109.69
**QCL**	1	23587.3	23112	97.98	22830.3	96.79	22907.3	97.12	25834.3	109.53
**Long-term Stability**										
		0 week	1 week		2 weeks		3 weeks		4 weeks	
**QCH**	7	230747.3	229995.3	99.67	238324.3	103.28	237654.3	102.99	244683	100.13
**QCM**	4	127275	119545	93.93	123021.3	96.66	128425	100.90	121302.7	95.56
**QCL**	1	28017.3	26697.7	95.29	27450.3	97.98	27808	99.25	25792.3	94.34

*QCH = quality control high, QCM = quality control medium, QCL = quality control low.

Long-term stability studies at −20 ± 5 °C over four weeks demonstrated that QCH, QCM, and QCL samples maintained their accuracy of 100.13%, 95.56%, and 94.34%, respectively (Table 3).

These results collectively demonstrate that paclitaxel in plasma is stable under routine laboratory conditions, including short-term bench-top exposure, auto-sampler storage, repeated freeze–thaw cycles, and long-term storage at −20 °C (Table 3).

#### Matrix effect.

Matrix effect evaluation confirmed that the bioanalytical method performs consistently with little variation across different human plasma lots (Supp Table 6 in S1 File).

#### Dilution effect.

Dilution integrity studies showed that the concentration of the diluted samples remained within ±15% of the nominal values, with a coefficient of variation (COV%) below 15%, confirming the method’s reliability for samples requiring dilution (Supp Table 7 in S1 File).

#### Carry-over and selectivity.

Selectivity analysis revealed that there is no interference peaks at the retention time of paclitaxel peak (Fig 3). The method is selective and shows no interference with plasma constituents, with no carry-over effects observed (Supp Table 8 in S1 File).

#### Ruggedness.

Ruggedness was evaluated by two analysts performing assays under specific chromatographic conditions. Peak areas and calibration curves were consistent with regression coefficients 0.9994 and 0.9988, confirming method reproducibility (Supp Table 9 in S1 File).

#### Application of the method.

The proposed HPLC method was successfully applied to determine the plasma concentration of paclitaxel in six female breast cancer patients. A total of twelve samples were collected pre-dose and post-dose. The observed C_max_ and C_min_ depicted in Table 4, demonstrating the method’s suitability for clinical pharmacokinetic study.

**Table 4:** Clinical application of the validated HPLC method (n = 6).

Patient’s code	Age (years) *	Weight (kgs) *	Total dose* (mg)	C_min_ (μg/ml)	C_max_ (μg/ml)
P-001	49.17 ± 13.9	51.5 ± 5.39	112 ± 5.06	0.09	3.36
P-002				0.55	2.00
P-003				0.32	2.37
P-004				0.72	2.23
P-005				0.16	2.25
P-006				0.00	3.32
Mean±SD				0.31 ± 0.28	2.59 ± 0.59

*Values are mentioned in Mean±SD, Dose level of paclitaxel was 80 mg/m^2^ given as one hour IV infusion.

Tan et al. reported C_max_ as 2.38 μg/ml after an 80 mg/m^2^ 1-hr infusion [23]. Tomoko Ohtsu, 1995 documented 2.25 μg/ml following a 105 mg/m^2^ 3 hr infusion [24]. W T Lim et al., in 2009 reported C_max_ of 1.188 μg/ml (976.8–1644.6) following an 80 mg/m^2^ 1-hour paclitaxel infusion [25]. In another clinical trial, the reported C_max_ was 2.364 μg/ml after an 80 mg/m^2^ I-hour IV infusion [26]. However, in our study the mean C_max_ was 2.59 μg/ml with a standard deviation of 0.59 following an 80 mg/m^2^ paclitaxel dose (Table 4). The determined values of C_max_ found comparable with these reports.

#### Greenness assessment of the method.

A new strategy called “green analytical chemistry” (GAC) seeks to reduce the negative effects of analytical chemistry on the environment and human health [27]. It places a strong emphasis on the development of analytical techniques that are safer for people and more environmentally friendly, such as minimizing the waste, energy use, and hazardous chemicals/reagents. The quantity and toxicity of reagents, waste production, energy needs, the number of procedural steps, miniaturization, and automation are the factors that determine the greenness of analytical approach.

Multiple parameter-based metric tools, which offer accurate and thorough evaluations of the greenness of analytical techniques, were used to precisely analyze the greenness levels of the HPLC method and determine how well it satisfies the requirements of GAC principles. These metrics included the Analytical Greenness (AGREE), AGREEprep, Complex Green Analytical Procedure Index (ComplexGAPI), Analytical Method Volume Intensity (AMVI), Analytical Eco-scale (ESA), and Analytical Method Greenness Score (AMGS) [28–33].

The most popular quantitative method for evaluating greenness is the AGREE technique. This methodology is based on 12 green chemistry principles. A score above 0.5 is considered the greenness of the method [28]. As a result, weights were given to criteria according to their significance, with a focus on simplicity, sample size, sample throughput, and energy consumption. Excellent greenness is indicated by an AGREE score of ≥ 0.75, appropriate greenness is indicated by a score between 0.74 and 0.50, and poor greenness is indicated by a score more than 0.50 [28]. The environmental sustainability of the proposed HPLC method was evaluated using this tool. As mentioned in Fig 4(a), the suggested method’s overall score was 0.57, indicating an appropriate degree of greenness.

**Fig 4:**
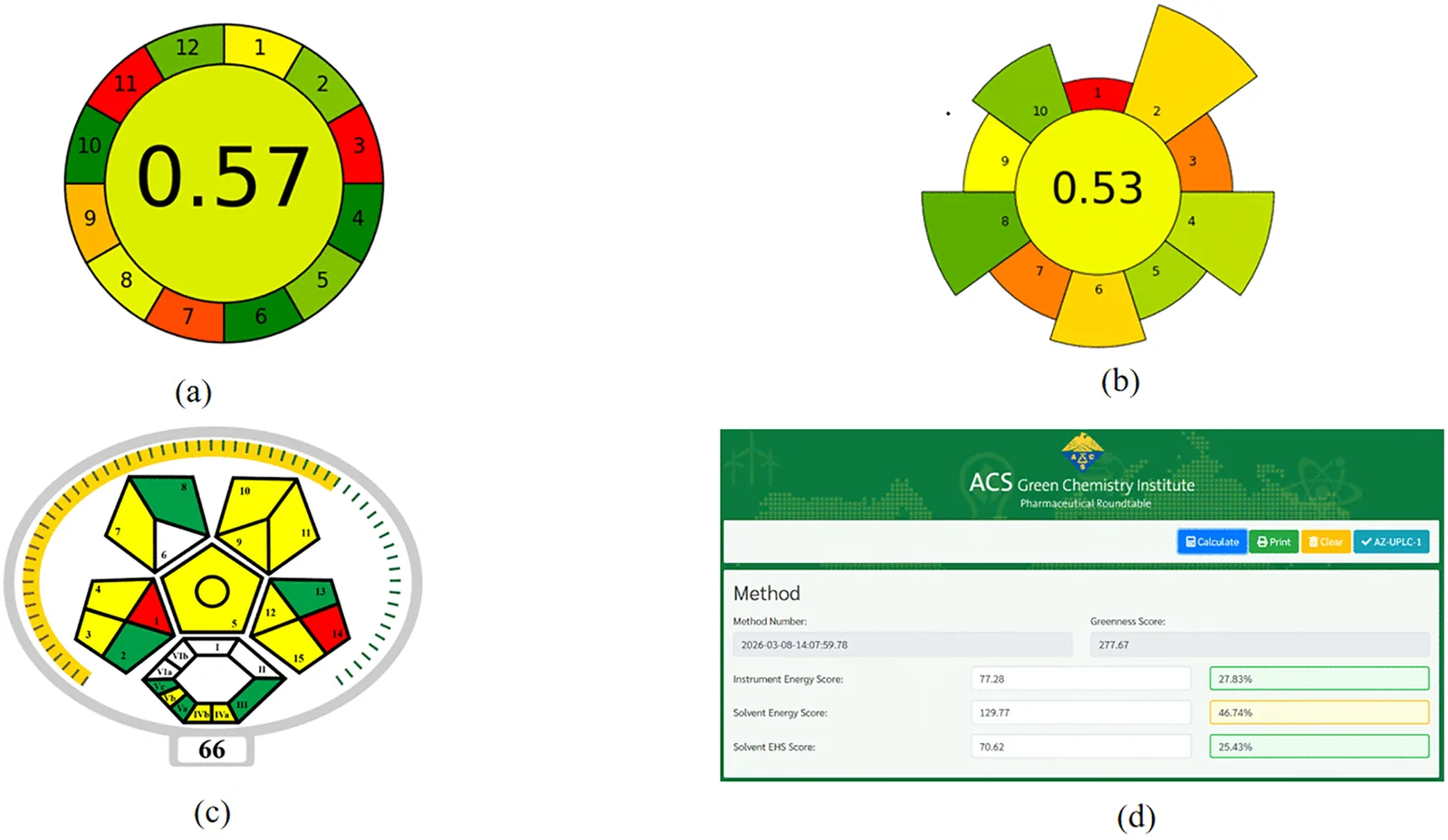
Greenness assessment of the proposed method by AGREE (a), AGREEprep (b), ComplexGAPI (c), AMGS (d).

The AGREEprep measure is utilized in analytical procedures to evaluate how sample preparation techniques affect the environment and analyze 10 GAC principles [29]. Each principle was given the same weight as those of the AGREE tool, resulting in the AGREEprep pictogram displayed in Fig 4(b) and a score of 0.53, which indicates that the sample preparation procedure was reasonably green.

GAPI was proposed by Płotka-Wasylka et al. in 2018 [34]. The greenness of different stages of the entire analytical assay can be evaluated and revealed by the five pentagrams of the GAPI pictogram. Płotka-Wasylka and Wojnowski improved GAPI and proposed ComplexGAPI in 2021 [30]. The ComplexGAPI tool assesses the greenness of particular analytical techniques using 15 criteria. It combines the precise total score of the analytical Eco-Scale with the benefits of the GAPI pictogram’s visual impact [30]. This score divides the approaches into three categories: inadequate (<50), acceptable (50–74), and exceptional (≥75). We used this new tool for evaluation of our proposed method. Fig 4(c) shows an acceptable greenness score of 66 for the suggested method. The use of low-toxicity reagents and conventional HPLC equipment improved the low-impact, sustainable strategy in our situation, supporting its designation as such.

R. Hartman et al. presented the AMVI green metric approach in 2011 [31]. The metric is a quantitative instrument intended for the HPLC procedure. Waste produced during HPLC use and solvent consumption are taken into account while calculating the %AMVI value. The ratio of total solvent consumption to the number of chromatographic peaks of interest is the AMVI value. The proposed method has an AMVI 131 score depicting its environmental friendliness.

When evaluating sustainability, the ESA measure must be used. A penalty score is determined by factors such as the quantity of energy used, the amount of waste produced, the possibility of hazards, and the use of reagents. A comprehensive sustainability level is calculated by adding these scores [32]. A system is categorized as “subpar green” if it receives less than 50 points, “superior green” if it receives more than 75 points, or “ideal green” if it receives between 50 and 75 points. With a score of 83, the method is ecologically sustainable and environmentally friendly.

AMGS was suggested as a new GAC metric by Hicks et al. [33] to assess and compare different approaches for greenness. Better greenness in the test is indicated by a lower score. AMGS was used to calculate the greenness score of the proposed method and found 277.67, indicating moderate energy demands and a balanced solvent-related environmental, health, and safety (EHS) profile, making it suitable for routine use in environmentally conscious laboratories as in Fig 4(d).

## Discussion

Pharmacokinetic and pharmacodynamic investigations play a crucial role in drug development by enabling accurate quantification of drugs in biological matrices. HPLC remains a preferable method due to its superior sensitivity, selectivity, cost-effectiveness, and accessibility. The method developed in this study was validated as per ICH M10 and FDA guidelines [21,22].

The proposed method demonstrated excellent linearity, with a detectable concentration of paclitaxel below 0.375 μg/ml. The extraction efficiency was consistently above 90%, with a COV of less than 2%. The results were consistent with previously reported reference methodologies, indicating reliable measurement of paclitaxel in plasma samples [16,17]. The LLOQ for paclitaxel was 0.375 μg/ml, which is comparable with previous reports [35]. LLOQ is slightly higher than some reported methods [12,15,18], however, those methods involve complex and expensive techniques, like LC-MS, a high proportion of organic solvents, and solid-phase extraction. However, this method is simple and quick, with a run time of only 10 min.

C_max_ of paclitaxel ranges from 2.6 to 13.9 μg/ml for the dose range of 105−270 mg/m^2^ over a 3-hr infusion, as reported in previous clinical studies [24,36]. The developed method reliability detects these concentrations with <20% COV, confirming its suitability for pharmacokinetic study in breast cancer patients. Blood samples were collected from patients with breast cancer in accordance with the institutional guidelines (IRB Ref. No: IRB-4026/DUHS/Approval/2025/307, dated: August 12, 2025). Plasma was separated from blood samples and, before analysis, stored at −20°C. The paclitaxel concentrations (C_max_ and C_min_) were determined using the developed method and found comparable with the existing literature [24]. The suggested HPLC method’s LLOQ is greater than that of LC-MS/MS techniques, but it was still adequate to measure the concentration of paclitaxel in the clinical samples examined in this investigation.

Overall, the validated HPLC method demonstrates satisfactory performance in accordance with international regulatory guidelines [21,22]. It exhibited excellent linearity over the defined calibration range, with a correlation coefficient (R^2^ = 0.9999). The sensitivity result established an acceptable LLOQ, ensuring reliable detection of paclitaxel at a low concentration level. Recovery studies show consistent and reproducible extraction efficiency across all QC levels. Accuracy and precision of inter-day and intra-day were within the predefined acceptable limits (±15% for calibrators and QCs and ±20% for LLOQ) confirming the method’s reliability and reproducibility. Paclitaxel’s stability under the tested conditions was confirmed by the slightly elevated freeze-thaw stability values (105–109%), which probably reflect typical analytical variability during sample processing and remain within the ± 15% acceptance limits recommended by FDA and ICH bioanalytical validation guidelines [21,22].

The matrix effects were assessed by selectivity and interference, indicated no significant interference from endogenous plasma components, confirming this method’s selectivity and robustness. However, future research will take into account a thorough matrix factor evaluation employing several plasma batches. Dilution integrity and carryover met the acceptance criteria. Overall, all validation parameters complied with the regulatory standards [21,22]. Therefore, it was concluded that the proposed HPLC method is accurate, precise, robust, and reliable for routine quantification of paclitaxel in human plasma samples.

The proposed HPLC method accurately determined the concentration of paclitaxel in female breast cancer patients. The C_max_ values are consistent with the reported values in literature. The development and validation of HPLC methods is the main focus of this initial pilot project. Because comprehensive pharmacokinetic parameters were beyond the scope of this investigation, the pharmacokinetic application was restricted to C_max_ and C_min_ determination.

The proposed method’s greenness was assessed by utilizing different metrics. The AGREE, AGREEprep, and ComplexGAPI scores confirmed that the method proposed is environmental friendly, while maintaining analytical efficiency. AMVI, ESA, and AMGS values showed balanced solvent-related environmental, health, and safety (EHS) profiles and moderate energy demands, making it appropriate for regular usage in laboratories.

The proposed method was developed as a straightforward and affordable assay for regular therapeutic drug monitoring, even though an internal standard is typically advised to account for extraction variability, injection errors, and matrix effects. The lack of an internal standard did not affect assay reliability, since method validation showed satisfactory linearity, accuracy, precision, recovery, selectivity, and stability. However, adding an appropriate internal standard could enhance method resilience even more, and this will be taken into account in future studies.

## Conclusion

The proposed HPLC method provides a simple, rapid, and robust approach for the quantification of paclitaxel in human plasma. The method demonstrated excellent linearity, precision, and accuracy, involving a single-step extraction with no interference from plasma constituents. The LLOQ of 0.375 μg/ml is sufficiently low to detect clinically relevant paclitaxel’s concentration. The method was found fully validated following FDA and ICH guidelines. Different greenness evaluation metrics confirmed the greenness of the proposed method. Overall, this method is accurate, precise, reproducible, and suitable for routine clinical pharmacokinetic and bioanalytical applications.

## Supporting information

S1 File Supplementary tables and figures. (DOC)
