# Supplementary material for: Development and clinical validation of HPLC method for precise quantification of paclitaxel in plasma from breast cancer patients
Source: PLoS One. 2026 Oct 8;21(10):e0358891. doi: 10.1371/journal.pone.0358891 (PMC13649134; doi:10.1371/journal.pone.0358891)
Supplement: Supplementary file 1 [file pone.0358891.s001.doc]

**Supplementary File**

**Development and clinical validation of HPLC method for precise quantification of paclitaxel in plasma from breast cancer patients**

Najia Rahim^1*^, Sadia Shakeel^1^, Maryum Nouman^2^, Sadia Asim^3^, Javeria Chaudhry^3^, Zulqarnain Hassan^3^.

^1^Department	of Pharmacy Practice, Dow College of Pharmacy,

Dow University of Health Sciences, Karachi, Pakistan.

^2^Department of Medical Oncology, Dow University Hospital,

Karachi, Pakistan.

^3^Dow Institute of Biological, Biochemical & Pharmaceutical Sciences, Dow University of Health Sciences, Karachi, Pakistan.

*Corresponding author: [najia.rahim@duhs.edu.pk](mailto:najia.rahim@duhs.edu.pk)

**Short title:** Development and clinical validation of HPLC method for precise quantification

**Supp. Table 1.** System suitability and linearity results.

| **Conc. added** | **Peak area** | **Retention time** | **No. theoretical plates** | **Tailing factor** | **Conc. calc.** | **% error** |
| --- | --- | --- | --- | --- | --- | --- |
| 8 | 297570 | 6.461 | 3896 | 1.787 | 7.99 | 99.90 |
| 6 | 223164 | 6.458 | 3900 | 1.714 | 6.01 | 100.38 |
| 5 | 185914 | 6.469 | 3862 | 1.638 | 5.03 | 100.25 |
| 2.5 | 88660 | 6.454 | 3767 | 1.545 | 2.45 | 97.75 |
| 1.5 | 52539 | 6.459 | 3669 | 1.437 | 1.49 | 98.90 |
| 0.75 | 26107 | 6.457 | 3961 | 1.286 | 0.79 | 103.19 |
| 0.375 | 10209 | 6.47 | 3967 | 1.365 | 0.37 | 105.55 |
| Linear equation (correlation R^2^) | | | | | Y = 37717x - 3681.3 (0.9999) | |

**Supp. Figure 1.** HPLC chromatograms of paclitaxel spiked in drug-free plasma from humans (concentration ranges 8-0.375 μg/ml).

**Supp. Table 2.** Sensitivity analysis results.

| **Conc. added**  **(ug/ml)** | **Peak area** | **Conc. calc.**  **(ug/ml)** | **Accuracy**  **(%)** |
| --- | --- | --- | --- |
| 8 | 242835 | 8.06 | 100.73 |
| 6 | 179567 | 5.99 | 99.85 |
| 5 | 146619 | 4.91 | 98.30 |
| 2.5 | 72406 | 2.49 | 99.60 |
| 1.5 | 42398 | 1.51 | 100.63 |
| 0.75 | 19798 | 0.77 | 102.81 |
| 0.375 | 8136 | 0.39 | 104.00 |
| 0.375 | 7188 | 0.36 | 95.74 |
| 0.375 | 7031 | 0.35 | 94.37 |
| 0.375 | 7083 | 0.36 | 94.83 |
| 0.375 | 7031 | 0.35 | 94.37 |
| 0.375 | 7137 | 0.36 | 95.30 |
| 0.375 | 7380 | 0.37 | 97.41 |
| 0.375 | 7187 | 0.36 | 95.73 |
| 0.375 | 7132 | 0.36 | 95.25 |
| 0.375 | 7129 | 0.36 | 95.23 |
| 0.375 | 7170 | 0.36 | 95.58 |
| 0.375 | 7198 | 0.36 | 95.83 |
| 0.375 | 7112 | 0.36 | 95.08 |
| 0.375 | 7059 | 0.35 | 94.62 |
| 0.375 | 7099 | 0.36 | 94.96 |
| 0.375 | 7141 | 0.36 | 95.33 |

**Supp Table 3**. Stock solution stability results.

| **Stock Solution Stability** | | | | | |
| --- | --- | --- | --- | --- | --- |
| **Conc. added**  **(ug/ml)** | **Peak area**  **(week 0)** | **Mean peak area**  **(A)** | **Peak area**  **(week 1)** | **Mean peak area**  **(B)** | **Accuracy %**  **Y=B/A*100** |
| 100 | 7045528 | 7056489.3 | 6801661 | 6894430.7 | 97.70 |
|  | 7057290 |  | 6959523 |  |  |
|  | 7066650 |  | 6922108 |  |  |
| **Conc. added**  **(ug/ml)** | **Peak area**  **(week 0)** | **Mean peak area**  **(A)** | **Peak area**  **(week 2)** | **Mean peak area**  **(B)** | **Accuracy %**  **Y=B/A*100** |
| 100 | 7045528 | 7056489.3 | 7338254 | 7348593.3 | 104.14 |
|  | 7057290 |  | 7342737 |  |  |
|  | 7066650 |  | 7364789 |  |  |
| **Conc. added**  **(ug/ml)** | **Peak area**  **(week 0)** | **Mean peak area**  **(A)** | **Peak area**  **(week 3)** | **Mean peak area**  **(B)** | **Accuracy %**  **Y=B/A*100** |
| 100 | 7045528 | 7056489.3 | 7085465 | 7122486.7 | 100.94 |
| 100 | 7057290 |  | 7145173 |  |  |
| 100 | 7066650 |  | 7136822 |  |  |
| **Conc. added**  **(ug/ml)** | **Peak area**  **(week 0)** | **Mean peak area**  **(A)** | **Peak area**  **(week 4)** | **Mean peak area**  **(B)** | **Accuracy %**  **Y=B/A*100** |
| 100 | 7045528 | 7056489.3 | 7352621 | 7419697.3 | 105.15 |
|  | 7057290 |  | 7427610 |  |  |
|  | 7066650 |  | 7478861 |  |  |

**Supp Table 4.** Auto-sampler stability results.

| **AUTO SAMPLER STABILITY**  **QCH*** | | | | | | | | | | |  |
| --- | --- | --- | --- | --- | --- | --- | --- | --- | --- | --- | --- |
| **Conc. added**  **(ug/ml)** | | **Peak area**  **(hour 0)** | | **Mean peak area**  **(A)** | | **Peak area**  **(hour 3)** | | **Mean**  **peak area**  **(B)** | **Accuracy %**  **Y=B/A*100** | |  |
| 7 | | 221293 | | 223411.0 | | 221066 | | 222785.3 | 99.72 | |  |
|  |  | 222042 | |  |  | 225275 | |  |  |  |  |
|  |  | 226898 | |  |  | 222015 | |  |  |  |  |
| **Conc. added**  **(ug/ml)** | | **Peak area**  **(hour 0)** | | **Mean peak area**  **(A)** | | **Peak area**  **(hour 6)** | | **Mean peak area**  **(B)** | **Accuracy %**  **Y=B/A*100** | |  |
| 7 | | 221293 | | 223411.0 | | 226743 | | 222093.3 | 99.41 | |  |
|  |  | 222042 | |  |  | 221686 | |  |  |  |  |
|  |  | 226898 | |  |  | 217851 | |  |  |  |  |
| **Conc. added**  **(ug/ml)** | | **Peak area**  **(hour 0)** | | **Mean peak area**  **(A)** | | **Peak area**  **(hour 10)** | | **Mean peak area**  **(B)** | **Accuracy %**  **Y=B/A*100** | |  |
| 7 | | 221293 | | 223411.0 | | 225277 | | 224282.7 | 100.39 | |  |
|  |  | 222042 | |  |  | 224010 | |  |  |  |  |
|  |  | 226898 | |  |  | 223561 | |  |  |  |  |
| **Conc. added**  **(ug/ml)** | | **Peak area**  **(hour 0)** | | **Mean peak area**  **(A)** | | **Peak area**  **(hour 24)** | | **Mean peak area**  **(B)** | **Accuracy %**  **Y=B/A*100** | |  |
| 7 | | 221293 | | 223411.0 | | 215376 | | 215158.3 | 96.31 | |  |
|  |  | 222042 | |  |  | 215658 | |  |  |  |  |
|  |  | 226898 | |  |  | 214441 | |  |  |  |  |
| **AUTO SAMPLER STABILITY**  **QCM** | | | | | | | | | | | |
| **Conc. added**  **(ug/ml)** | **Peak area**  **(hour 0)** | | **Mean peak area**  **(A)** | | **Peak area**  **(hour 3)** | | **Mean peak area**  **(B)** | | | **Accuracy %**  **Y=B/A*100** | |
| 4 | 115203 | | 118288.0 | | 115264 | | 116753.3 | | | 98.70 | |
|  | 119127 | |  |  | 118093 | |  |  |  |  |  |
|  | 120534 | |  |  | 116903 | |  |  |  |  |  |
| **Conc. added**  **(ug/ml)** | **Peak area**  **(hour 0)** | | **Mean peak area**  **(A)** | | **Peak area**  **(hour 6)** | | **Mean peak area**  **(B)** | | | **Accuracy %**  **Y=B/A*100** | |
| 4 | 115203 | | 118288.0 | | 122193 | | 120910.0 | | | 102.22 | |
|  | 119127 | |  |  | 120293 | |  |  |  |  |  |
|  | 120534 | |  |  | 120244 | |  |  |  |  |  |
| **Conc. added**  **(ug/ml)** | **Peak area**  **(hour 0)** | | **Mean peak area**  **(A)** | | **Peak area**  **(hour 10)** | | **Mean peak area**  **(B)** | | | **Accuracy %**  **Y=B/A*100** | |
| 4 | 115203 | | 118288.0 | | 121739 | | 120017.7 | | | 101.46 | |
|  | 119127 | |  |  | 118294 | |  |  |  |  |  |
|  | 120534 | |  |  | 120020 | |  |  |  |  |  |
| **Conc. added**  **(ug/ml)** | **Peak area**  **(hour 0)** | | **Mean peak area**  **(A)** | | **Peak area**  **(hour 24)** | | **Mean peak area**  **(B)** | | | **Accuracy %**  **Y=B/A*100** | |
| 4 | 115203 | | 118288.0 | | 113888 | | 115015.7 | | | 97.23 | |
|  | 119127 | |  |  | 114403 | |  |  |  |  |  |
|  | 120534 | |  |  | 116756 | |  |  |  |  |  |
| **AUTO SAMPLER STABILITY**  **QCL** | | | | | | | | | | | |
| **Conc. added**  **(ug/ml)** | **Peak area**  **(hour 0)** | | **Mean peak area**  **(A)** | | **Peak area**  **(hour 3)** | | **Mean peak area**  **(B)** | | | **Accuracy %**  **Y=B/A*100** | |
| 1 | 26066 | | 25780.7 | | 25104 | | 25370.0 | | | 98.41 | |
|  | 25820 | |  |  | 25516 | |  |  |  |  |  |
|  | 25456 | |  |  | 25490 | |  |  |  |  |  |
| **Conc. added**  **(ug/ml)** | **Peak area**  **(hour 0)** | | **Mean peak area**  **(A)** | | **Peak area**  **(hour 6)** | | **Mean peak area**  **(B)** | | | **Accuracy %**  **Y=B/A*100** | |
| 1 | 26066 | | 25780.7 | | 26952 | | 26073.7 | | | 101.14 | |
|  | 25820 | |  |  | 25471 | |  |  |  |  |  |
|  | 25456 | |  |  | 25798 | |  |  |  |  |  |
| **Conc. added**  **(ug/ml)** | **Peak area**  **(hour 0)** | | **Mean peak area**  **(A)** | | **Peak area**  **(hour 10)** | | **Mean peak area**  **(B)** | | | **Accuracy %**  **Y=B/A*100** | |
| 1 | 26066 | | 25780.7 | | 26770 | | 26799.7 | | | 103.95 | |
|  | 25820 | |  |  | 27504 | |  |  |  |  |  |
|  | 25456 | |  |  | 26125 | |  |  |  |  |  |
| **Conc. added**  **(ug/ml)** | **Peak area**  **(hour 0)** | | **Mean peak area**  **(A)** | | **Peak area**  **(hour 24)** | | **Mean peak area**  **(B)** | | | **Accuracy %**  **Y=B/A*100** | |
| 1 | 26066 | | 25780.7 | | 24241 | | 24754.0 | | | 96.02 | |
|  | 25820 | |  |  | 24325 | |  |  |  |  |  |
|  | 25456 | |  |  | 25696 | |  |  |  |  |  |

#### *QCH = quality control high, QCM = quality control medium, QCL = quality control low.

**Supp Table 5.** Bench-top stability results.

| **BENCHTOP STABILITY**  **QCH*** | | | | | | | | | | |
| --- | --- | --- | --- | --- | --- | --- | --- | --- | --- | --- |
| **Conc. added**  **(ug/ml)** | | **Peak area**  **(hour 0)** | | **Mean peak area**  **(A)** | | **Peak area**  **(hour 3)** | | **Meanpeak area**  **(B)** | | **Accuracy %**  **Y=B/A*100** |
| 7 | | 215872 | | 216590.3 | | 220572 | | 218229.7 | | 100.76 |
|  |  | 216341 | |  |  | 218694 | |  |  |  |
|  |  | 217558 | |  |  | 215423 | |  |  |  |
| **Conc. added**  **(ug/ml)** | | **Peak area**  **(hour 0)** | | **Meanpeak area**  **(A)** | | **Peak area**  **(hour 6)** | | **Mean peak area**  **(B)** | | **Accuracy %**  **Y=B/A*100** |
| 7 | | 215872 | | 216590.3 | | 227445 | | 221116.7 | | 102.09 |
|  |  | 216341 | |  |  | 219556 | |  |  |  |
|  |  | 217558 | |  |  | 216349 | |  |  |  |
| **Conc. added**  **(ug/ml)** | | **Peak area**  **(hour 0)** | | **Mean peak area**  **(A)** | | **Peak area**  **(hour 10)** | | **Mean peak area**  **(B)** | | **Accuracy %**  **Y=B/A*100** |
| 7 | | 215872 | | 216590.3 | | 215304 | | 218467.3 | | 100.87 |
|  |  | 216341 | |  |  | 219293 | |  |  |  |
|  |  | 217558 | |  |  | 220805 | |  |  |  |
| **Conc. added**  **(ug/ml)** | | **Peak area**  **(hour 0)** | | **Mean peak area**  **(A)** | | **Peak area**  **(hour 24)** | | **Mean peak area**  **(B)** | | **Accuracy %**  **Y=B/A*100** |
| 7 | | 215872 | | 216590.3 | | 210960 | | 210770.7 | | 97.31 |
|  |  | 216341 | |  |  | 211348 | |  |  |  |
|  |  | 217558 | |  |  | 210004 | |  |  |  |
| **BENCHTOP STABILITY**  **QCM** | | | | | | | | | | |
| **Conc. added**  **(ug/ml)** | **Peak area**  **(hour 0)** | | **Mean**  **peak area**  **(A)** | | **Peak area**  **(hour 3)** | | **Mean**  **peak area**  **(B)** | | **Accuracy %**  **Y=B/A*100** | |
| 4 | 118297 | | 116676.0 | | 117683 | | 116386.0 | | 99.75 | |
|  | 116378 | |  |  | 115841 | |  |  |  |  |
|  | 115353 | |  |  | 115634 | |  |  |  |  |
| **Conc. added**  **(ug/ml)** | **Peak area**  **(hour 0)** | | **Mean peak area**  **(A)** | | **Peak area**  **(hour 6)** | | **Mean peak area**  **(B)** | | **Accuracy %**  **Y=B/A*100** | |
| 4 | 118297 | | 116676.0 | | 119057 | | 118157.0 | | 101.27 | |
|  | 116378 | |  |  | 119429 | |  |  |  |  |
|  | 115353 | |  |  | 115985 | |  |  |  |  |
| **Conc. added**  **(ug/ml)** | **Peak area**  **(hour 0)** | | **Mean peak area**  **(A)** | | **Peak area**  **(hour 10)** | | **Mean peak area**  **(B)** | | **Accuracy %**  **Y=B/A*100** | |
| 4 | 118297 | | 116676.0 | | 121091 | | 121551.7 | | 104.18 | |
|  | 116378 | |  |  | 121654 | |  |  |  |  |
|  | 115353 | |  |  | 121910 | |  |  |  |  |
| **Conc. added**  **(ug/ml)** | **Peak area**  **(hour 0)** | | **Mean peak area**  **(A)** | | **Peak area**  **(hour 24)** | | **Mean peak area**  **(B)** | | **Accuracy %**  **Y=B/A*100** | |
| 4 | 118297 | | 116676.0 | | 116559 | | 114373.7 | | 98.03 | |
|  | 116378 | |  |  | 113517 | |  |  |  |  |
|  | 115353 | |  |  | 113045 | |  |  |  |  |
| **BENCHTOP STABILITY**  **QCL** | | | | | | | | | | |
| **Conc. added**  **(ug/ml)** | **Peak area**  **(hour 0)** | | **Mean peak area**  **(A)** | | **Peak area**  **(hour 3)** | | **Mean peak area**  **(B)** | | **Accuracy %**  **Y=B/A*100** | |
| 1 | 26662 | | 26784.0 | | 26086 | | 25863.7 | | 96.56 | |
|  | 27035 | |  |  | 25625 | |  |  |  |  |
|  | 26655 | |  |  | 25880 | |  |  |  |  |
| **Conc. added**  **(ug/ml)** | **Peak area**  **(hour 0)** | | **Mean peak area**  **(A)** | | **Peak area**  **(hour 6)** | | **Mean peak area**  **(B)** | | **Accuracy %**  **Y=B/A*100** | |
| 1 | 26662 | | 26784.0 | | 26058 | | 25803.0 | | 96.34 | |
|  | 27035 | |  |  | 25661 | |  |  |  |  |
|  | 26655 | |  |  | 25690 | |  |  |  |  |
| **Conc. added**  **(ug/ml)** | **Peak area**  **(hour 0)** | | **Mean peak area**  **(A)** | | **Peak area**  **(hour 10)** | | **Mean peak area**  **(B)** | | **Accuracy %**  **Y=B/A*100** | |
| 1 | 26662 | | 26784.0 | | 26352 | | 26171.7 | | 97.71 | |
|  | 27035 | |  |  | 26272 | |  |  |  |  |
|  | 26655 | |  |  | 25891 | |  |  |  |  |
| **Conc. added**  **(ug/ml)** | **Peak area**  **(hour 0)** | | **Mean peak area**  **(A)** | | **Peak area**  **(hour 24)** | | **Mean peak area**  **(B)** | | **Accuracy %**  **Y=B/A*100** | |
| 1 | 26662 | | 26784.0 | | 25932 | | 24392.7 | | 91.07 | |
|  | 27035 | |  |  | 23774 | |  |  |  |  |
|  | 26655 | |  |  | 23472 | |  |  |  |  |

#### *QCH = quality control high, QCM = quality control medium, QCL = quality control low.

**Supp Table 6.** Matrix effect results.

| **S. No** | **QCs*** | **Peak area** | **Mean Peak**  **area** | **Conc. Cal.**  **(ug/ml)** | **Accuracy (%)** | **STD Dev.** | **Mean Conc.** | **%COV** |
| --- | --- | --- | --- | --- | --- | --- | --- | --- |
| 1 | QCH 7 | 216492 | 218873.67 | 7.43 | 106.10 | 0.02 | 7.51 | 0.33 |
|  | QCH 7 | 217527 |  | 7.46 | 106.59 |  |  |  |
|  | QCH 7 | 222602 |  | 7.63 | 109.01 |  |  |  |
|  | QCL 1 | 22727 | 22643.00 | 0.96 | 95.88 | 0.01 | 0.96 | 0.75 |
|  | QCL 1 | 22424 |  | 0.95 | 94.87 |  |  |  |
|  | QCL 1 | 22778 |  | 0.96 | 96.05 |  |  |  |
| 2 | QCH 7 | 208168 | 208365.33 | 7.15 | 102.13 | 0.01 | 7.16 | 0.17 |
|  | QCH 7 | 208682 |  | 7.17 | 102.37 |  |  |  |
|  | QCH 7 | 208246 |  | 7.15 | 102.17 |  |  |  |
|  | QCL 1 | 22599 | 22621.67 | 0.95 | 95.45 | 0.00 | 0.96 | 0.19 |
|  | QCL 1 | 22524 |  | 0.95 | 95.20 |  |  |  |
|  | QCL 1 | 22742 |  | 0.96 | 95.93 |  |  |  |
| 3 | QCH 7 | 209037 | 208772.33 | 7.18 | 102.54 | 0.02 | 7.17 | 0.31 |
|  | QCH 7 | 209971 |  | 7.21 | 102.99 |  |  |  |
|  | QCH 7 | 207309 |  | 7.12 | 101.72 |  |  |  |
|  | QCL 1 | 22663 | 22577.00 | 0.96 | 95.67 | 0.01 | 0.95 | 0.78 |
|  | QCL 1 | 22347 |  | 0.95 | 94.61 |  |  |  |
|  | QCL 1 | 22721 |  | 0.96 | 95.86 |  |  |  |
| 4 | QCH 7 | 212505 | 211854.33 | 7.29 | 104.20 | 0.02 | 7.27 | 0.27 |
|  | QCH 7 | 211675 |  | 7.27 | 103.80 |  |  |  |
|  | QCH 7 | 211383 |  | 7.26 | 103.66 |  |  |  |
|  | QCL 1 | 22970 | 22898.67 | 0.97 | 96.69 | 0.01 | 0.96 | 0.71 |
|  | QCL 1 | 22680 |  | 0.96 | 95.73 |  |  |  |
|  | QCL 1 | 23046 |  | 0.97 | 96.95 |  |  |  |
| 5 | QCH 7 | 213904 | 212472.67 | 7.34 | 104.87 | 0.05 | 7.29 | 0.66 |
|  | QCH 7 | 211858 |  | 7.27 | 103.89 |  |  |  |
|  | QCH 7 | 211656 |  | 7.27 | 103.79 |  |  |  |
|  | QCL 1 | 22559 | 22520.67 | 0.95 | 95.32 | 0.01 | 0.95 | 0.72 |
|  | QCL 1 | 22269 |  | 0.94 | 94.35 |  |  |  |
|  | QCL 1 | 22734 |  | 0.96 | 95.91 |  |  |  |
| 6 | QCH 7 | 211618 | 212393.33 | 7.26 | 103.78 | 0.06 | 7.29 | 0.82 |
|  | QCH 7 | 214149 |  | 7.35 | 104.98 |  |  |  |
|  | QCH 7 | 211413 |  | 7.26 | 103.68 |  |  |  |
|  | QCL 1 | 23186 | 23396.00 | 0.97 | 97.41 | 0.01 | 0.98 | 1.01 |
|  | QCL 1 | 23605 |  | 0.99 | 98.81 |  |  |  |
|  | QCL 1 | 23397 |  | 0.98 | 98.12 |  |  |  |

#### *QCs = quality control samples, QCH = quality control high, QCL = quality control low.

Supp Table 7. Dilution integrity results.

| **Dilution** | **Peak area** | **Conc. calc.**  **(ug/ml)** | **Dilution factor** | **Accuracy (%)** | **Std. Dev.** | **Mean Conc.** | **COV%** |
| --- | --- | --- | --- | --- | --- | --- | --- |
| **16** | 488051 | 16.15 | **-** | 100.91 | 0.09 | 16.09 | 0.58 |
|  | 483030 | 15.98 | **-** | 99.87 |  |  |  |
|  | 487716 | 16.13 | **-** | 100.84 |  |  |  |
| **8** | 248334 | 8.20 | 02 | 102.47 | 0.02 | 8.20 | 0.29 |
|  | 249039 | 8.22 | 02 | 102.76 |  |  |  |
|  | 247615 | 8.17 | 02 | 102.17 |  |  |  |
| **4** | 115360 | 3.79 | 04 | 94.71 | 0.02 | 3.78 | 0.41 |
|  | 115061 | 3.78 | 04 | 94.46 |  |  |  |
|  | 114454 | 3.76 | 04 | 93.96 |  |  |  |

**Supp Table 8.** Carry-over effect results.

| **Conc.**  **added**  **(ug/ml)** | **Peak area in blank plasma** | **Peak area of drug** | **Carry over= Area in blank plasma/Area of Drug X 100** |
| --- | --- | --- | --- |
| 8 ug/ml | 0 | 235204 | 0 |
| 6 ug/ml | 0 | 171078 | 0 |
| 5 ug/ml | 0 | 144098 | 0 |

**Supp Table 9.** Ruggedness results.

| **Analyst 1** | | | |
| --- | --- | --- | --- |
| **Conc.**  **added**  **(ug/ml)** | **Peak area** | **Conc. calc.**  **(ug/ml)** | **Accuracy**  **(%)** |
| 8 | 256434 | 8.09 | 101.16 |
| 6 | 184200 | 5.87 | 97.89 |
| 5 | 156108 | 5.01 | 100.21 |
| 2.5 | 73864 | 2.48 | 99.36 |
| 1.5 | 41781 | 1.50 | 99.89 |
| 0.75 | 16857 | 0.73 | 97.70 |
| 0.375 | 7111 | 0.43 | 115.56 |

| **Analyst 2** | | | |
| --- | --- | --- | --- |
| **Conc.**  **added**  **(ug/ml)** | **Peak area** | **Conc. Cal.**  **(ug/ml)** | **Accuracy**  **%** |
|  |  |  |  |
| 8 | 251495 | 8.05 | 100.66 |
| 6 | 184790 | 5.97 | 99.53 |
| 5 | 152425 | 4.96 | 99.25 |
| 2.5 | 73009 | 2.49 | 99.42 |
| 1.5 | 40252 | 1.46 | 97.58 |
| 0.75 | 17638 | 0.76 | 101.12 |
| 0.375 | 7129 | 0.43 | 114.83 |
