# Supplementary material for: Development and clinical validation of HPLC method for precise quantification of paclitaxel in plasma from breast cancer patients
Source: PLoS One. 2026 Oct 8;21(10):e0358891. doi: 10.1371/journal.pone.0358891 (PMC13649134; doi:10.1371/journal.pone.0358891)
Supplement: Supplementary file 2 [file pone.0358891.s003.doc]

**Response to Reviewers**

**PONE-D-26-17670**

| **Reviewer #** | **Comments** | **Corrections** | **Justifications** |
| --- | --- | --- | --- |
| 1 | In bioanalytical HPLC assays, an internal standard is generally recommended to compensate for extraction variability, injection errors, and matrix effects. The authors should justify why no standard was used and discuss its possible impact on assay reliability and reproducibility. | “The proposed method was developed as a straightforward and affordable assay for regular therapeutic drug monitoring, even though an internal standard is typically advised to account for extraction variability, injection errors, and matrix effects. The lack of an internal standard did not affect assay reliability, since method validation showed satisfactory linearity, accuracy, precision, recovery, selectivity, and stability. However, adding an appropriate internal standard could enhance method resilience even more, and this will be taken into account in future studies.” | We appreciate the reviewer's insightful feedback. We recognize that in bioanalytical HPLC tests, the use of an internal standard (IS) is typically advised to account for matrix effects, injection mistakes, and extraction variability. If the method is thoroughly validated and satisfies the acceptance criteria for accuracy, precision, recovery, selectivity, and stability as specified in international bioanalytical validation guidelines, its usage is not required. Our approach showed satisfactory linearity, precision, accuracy, recovery, and stability, suggesting that assay repeatability and reliability were unaffected by the lack of an internal reference. However, we have highlighted this as a shortcoming in the updated publication and pointed out that future research could further improve method robustness by using an appropriate internal standard. |
|  | Important pharmacokinetic parameters such as AUC, half-life, clearance, and volume of distribution were not evaluated. The authors should either expand the PK analysis or clearly state that this is a preliminary pilot study. The pharmacokinetic application is restricted only to Cmax and Cmin determination in six patients. | “The development and validation of HPLC methods is the main focus of this initial pilot project. Because comprehensive pharmacokinetic parameters were beyond the scope of this investigation, the pharmacokinetic application was restricted to C_max_ and C_min_  determination.” | We appreciate the reviewer's insightful feedback. Rather than doing a thorough pharmacokinetic investigation, the main goal of this work was to design and clinically validate an HPLC method for paclitaxel quantification. Therefore, as a preliminary pilot research, the clinical application was restricted to C_max_ and C_min_ assessment in six individuals. This method will be utilized in our future research. The updated manuscript now makes this clear. |
|  | The clinical validation was conducted in only six breast cancer patients, which limits the statistical strength and generalizability of the findings. This limitation should be clearly acknowledged in the Discussion section. | Mentioned in discussion section. | We appreciate the reviewer's insightful feedback. We agree that the results' statistical strength and generalizability are limited by the small sample size. Larger clinical investigations are required to further validate the approach, as we have said in the Discussion section, that this is a pilot study. |
|  | The reported LLOQ (0.375 µg/mL) is higher than many previously reported LC-MS methods. The authors should better justify whether this sensitivity is sufficient for clinical pharmacokinetic and therapeutic monitoring applications. | “LLOQ is slightly higher than some reported methods , however, those methods involve complex and expensive techniques, like LC-MS, a high proportion of organic solvents, and solid-phase extraction. However, this method is simple and quick, with a run time of only 10 min.”  “The suggested HPLC method's LLOQ is greater than that of LC-MS/MS techniques, but it was still adequate to measure the concentration of paclitaxel in the clinical samples examined in this investigation.” | We appreciate the reviewer's insightful feedback. We agree that LC-MS/MS techniques typically yield lower LLOQs than HPLC-UV techniques. Nonetheless, the main goal of our research was to develop an easy-to-use, reasonably priced HPLC technique appropriate for standard clinical laboratories. Its applicability for the intended therapeutic drug monitoring application was demonstrated by the stated LLOQ of 0.375 µg/mL, which was sufficient to precisely quantify paclitaxel concentrations found in our clinical samples, including C_max_ and C_min_. The Discussion section now includes this explanation. |
|  | Important chromatographic details such as retention time, column particle size, column temperature, total run time, and system suitability parameters were not reported. These details are necessary for the reproducibility of the method. Although matrix effect evaluation was mentioned, detailed quantitative data and acceptance criteria were not adequately presented. The authors should provide matrix factor calculations and variability data from different plasma lots. | “Paclitaxel was analyzed using a C18 (5 µm, 250 mm × 4.6 mm) column at the detection wavelength of 227 nm. Column temperature and total run time were 40°C and 10 min.”  “The matrix effects were assessed by selectivity and interference, indicated no significant interference from endogenous plasma components, confirming this method’s selectivity and robustness. However, future research will take into account a thorough matrix factor evaluation employing several plasma batches. ” | Chromatographic details such as retention time and system suitability parameters are already mentioned in **Supp. Table 1.** System suitability and linearity results.  Matrix effect results are mentioned in  **Supp Table 6.**  We recognize that the current work did not undertake quantitative matrix factor calculations using several plasma batches. Since HPLC-UV detection was used to create the method, matrix effects were assessed by selectivity and interference tests, which showed no discernible endogenous interference at the paclitaxel retention duration. In the updated publication, we have acknowledged this restriction and stated that future research will take into account a more thorough matrix effect study. |
|  | Some freeze-thaw stability values exceeded 105-109%, which requires clarification. The authors should discuss the possible reasons for these variations and confirm compliance with FDA and ICH acceptance limits. | “Paclitaxel's stability under the tested conditions was confirmed by the slightly elevated freeze-thaw stability values (105–109%), which probably reflect typical analytical variability during sample processing and remain within the ±15% acceptance limits recommended by FDA and ICH bioanalytical validation guidelines (21, 22).” | We appreciate the reviewer's insightful feedback. Instead of analyte instability, the observed freeze-thaw stability values that are marginally higher than 100% (105–109%) are probably due to typical analytical variability related to sample preparation and instrument response. According to the FDA and ICH bioanalytical technique validation criteria, these results stay within the acceptable limits of ±15%, indicating that paclitaxel remained stable under the assessed freeze-thaw conditions. The updated Discussion section now includes this clarification. |
|  | Important patient-related information, such as inclusion-exclusion criteria, concurrent medications, hepatic or renal status, and infusion duration, was not adequately described. | “Female patients of age 18-75 years, diagnosed with breast cancer and prescribed with paclitaxel for chemotherapy were recruited from November’ 2025 to December’ 2025. Patients with pre-existing peripheral neuropathy, simultaneous anticancer treatment with hormone or immunotherapy, and severe hepatic/renal impairment were excluded.” | These information have been added in revised manuscript. |
|  | The authors are encouraged to provide raw data or validation datasets in a public repository or supplementary files. | ---- | We have provided raw data or validation datasets in the supplementary file. |
| 2 | The authors should add a high-resolution schematic illustration of the methodology and overall workflow to enhance the quality of the manuscript. | ---- | We appreciate the reviewer's insightful recommendation. Although we acknowledge that a schematic picture could improve the methodology's visual presentation, we think the Materials and Methods section currently provides enough information about the experimental approach. In order to prevent duplication and preserve the manuscript's clarity and conciseness, we have kept the current presentation. |
|  | Additionally, they should also provide high-resolution versions of the graphs, figures, and chromatograms. | Done | ---- |
|  | It is observed that the solvent used to prepare the paclitaxel stock solution has not been specified. Authors are required to mention the same. | “A primary stock solution was prepared by dissolving pure paclitaxel (10 mg) in 10 ml of methanol to obtain a concentration of 1 mg/ml.” | Already mentioned in method. |
|  | The clinical application was conducted in only six patients, which may be insufficient to conclude. Validation for a higher number of patients is recommended. | Mentioned in discussion section. | We appreciate the reviewer's insightful feedback. We agree that the results' statistical strength and generalizability are limited by the small sample size. Larger clinical investigations are required to further validate the approach, as we have said in the Discussion section, that this is a pilot study. |
|  | The authors are recommended to provide the raw chromatography data files, along with the analyzed images/data outputs, as supplementary information. | ---- | We have provided raw data or validation datasets in the supplementary file. |
|  | A comparison of the proposed method with traditional methods should be presented in a tabular format, covering runtime, solvent consumption, extraction efficiency, sensitivity, and cost-effectiveness. | ---- | We appreciate the reviewer's insightful recommendation. We have extended the Discussion section to compare the suggested approach with previously published HPLC methods in terms of runtime, solvent usage, sensitivity, and extraction efficiency rather than creating a separate comparison table. We have also emphasized that, in line with recent research, the suggested approach embodies the concepts of Green Analytical Chemistry (GAC) by decreasing the amount of solvent used and the duration of the analysis, which lessens the impact on the environment while preserving analytical performance. |
| Journal Requirements: | Please ensure that your manuscript meets PLOS ONE's style requirements, including those for file naming. | Done | ---- |
|  | Please state what role the funders took in the study.  If the funders had no role, please state: "The funders had no role in study design, data collection and analysis, decision to publish, or preparation of the manuscript."  If this statement is not correct you must amend it as needed.  Please include this amended Role of Funder statement in your cover letter; we will change the online submission form on your behalf. | Done | ---- |
|  | In the online submission form you indicate that your data is not available for proprietary reasons and have provided a contact point for accessing this data. Please note that your current contact point is a co-author on this manuscript. According to our Data Policy, the contact point must not be an author on the manuscript and must be an institutional contact, ideally not an individual. Please revise your data statement to a non-author institutional point of contact, such as a data access or ethics committee, and send this to us via return email. Please also include contact information for the third party organization, and please include the full citation of where the data can be found. | Done | ---- |
|  | Thank you for stating the following in the Acknowledgments Section of your manuscript:  “We are thankful to the Vice Chancellor, Dow University of Health Sciences, Professor Dr Nazli Hossain for the financial support (VCSFI 2024-25)”  We note that you have provided additional information within the Acknowledgements Section that is not currently declared in your Funding Statement. Please note that funding information should not appear in the Acknowledgments section or other areas of your manuscript. We will only publish funding information present in the Funding Statement section of the online submission form.  Please remove any funding-related text from the manuscript and let us know how you would like to update your Funding Statement. Currently, your Funding Statement reads as follows:  “VCSFI 2024-25 funding was received for this study. “  Please include your amended statements within your cover letter; we will change the online submission form on your behalf. | Done | ---- |
|  | Your ethics statement should only appear in the Methods section of your manuscript. If your ethics statement is written in any section besides the Methods, please move it to the Methods section and delete it from any other section. Please ensure that your ethics statement is included in your manuscript, as the ethics statement entered into the online submission form will not be published alongside your manuscript. | Done | ---- |
|  | If the reviewer comments include a recommendation to cite specific previously published works, please review and evaluate these publications to determine whether they are relevant and should be cited. There is no requirement to cite these works unless the editor has indicated otherwise. | ---- | Reviewer comments did not included any recommendation to cite specific previously published works. |
