# Supplementary material for: Development and clinical validation of HPLC method for precise quantification of paclitaxel in plasma from breast cancer patients
Source: PLoS One. 2026 Oct 8;21(10):e0358891. doi: 10.1371/journal.pone.0358891 (PMC13649134; doi:10.1371/journal.pone.0358891)
Supplement: Peer review history

# Peer review: Decision Letter 0

**DOI:** 10.1371/journal.pone.0358891.r001

**Authors:** Rajesh Kumar Singh

15 Jun 2026

Dear Dr. Rahim,

plosone@plos.org. When you're ready to submit your revision, log on to https://www.editorialmanager.com/pone/ and select the 'Submissions Needing Revision' folder to locate your manuscript file.

- A letter that responds to each point raised by the academic editor and reviewer(s). You should upload this letter as a separate file labeled 'Response to Reviewers'.
- A marked-up copy of your manuscript that highlights changes made to the original version. You should upload this as a separate file labeled 'Revised Manuscript with Track Changes'.
- An unmarked version of your revised paper without tracked changes. You should upload this as a separate file labeled 'Manuscript'.

If you would like to make changes to your financial disclosure, please include your updated statement in your cover letter. Guidelines for resubmitting your figure files are available below the reviewer comments at the end of this letter.

If applicable, we recommend that you deposit your laboratory protocols in protocols.io to enhance the reproducibility of your results. Protocols.io assigns your protocol its own identifier (DOI) so that it can be cited independently in the future. For instructions see: https://journals.plos.org/plosone/s/submission-guidelines#loc-laboratory-protocols. Additionally, PLOS ONE offers an option for publishing peer-reviewed Lab Protocol articles, which describe protocols hosted on protocols.io. Read more information on sharing protocols at https://plos.org/protocols?utm_medium=editorial-email&utm_source=authorletters&utm_campaign=protocols.

As the corresponding author, your ORCID iD is verified in the submission system and will appear in the published article. PLOS supports the use of ORCID, and we encourage all coauthors to register for an ORCID iD and use it as well. Please encourage your coauthors to verify their ORCID iD within the submission system before final acceptance, as unverified ORCID iDs will not appear in the published article. *Only*  the individual author can complete the verification step; PLOS staff *cannot*  verify ORCID iDs on behalf of authors.

We look forward to receiving your revised manuscript.

Kind regards,

Rajesh Kumar Singh, Ph.D.

Academic Editor

PLOS One

Journal Requirements:

When submitting your revision, we need you to address these additional requirements.

1. Please ensure that your manuscript meets PLOS ONE's style requirements, including those for file naming. The PLOS ONE style templates can be found at

https://journals.plos.org/plosone/s/file?id=wjVg/PLOSOne_formatting_sample_main_body.pdf and

https://journals.plos.org/plosone/s/file?id=ba62/PLOSOne_formatting_sample_title_authors_affiliations.pdf.

2. Thank you for stating the following financial disclosure:

“VCSFI 2024-25 funding was received for this study”

Please state what role the funders took in the study.  If the funders had no role, please state: "The funders had no role in study design, data collection and analysis, decision to publish, or preparation of the manuscript."

If this statement is not correct you must amend it as needed.

Please include this amended Role of Funder statement in your cover letter; we will change the online submission form on your behalf.

3. In the online submission form you indicate that your data is not available for proprietary reasons and have provided a contact point for accessing this data. Please note that your current contact point is a co-author on this manuscript. According to our Data Policy, the contact point must not be an author on the manuscript and must be an institutional contact, ideally not an individual. Please revise your data statement to a non-author institutional point of contact, such as a data access or ethics committee, and send this to us via return email. Please also include contact information for the third party organization, and please include the full citation of where the data can be found.

4. Thank you for stating the following in the Acknowledgments Section of your manuscript:

“We are thankful to the Vice Chancellor, Dow University of Health Sciences, Professor Dr Nazli Hossain for the financial support (VCSFI 2024-25)”

We note that you have provided additional information within the Acknowledgements Section that is not currently declared in your Funding Statement. Please note that funding information should not appear in the Acknowledgments section or other areas of your manuscript. We will only publish funding information present in the Funding Statement section of the online submission form.

Please remove any funding-related text from the manuscript and let us know how you would like to update your Funding Statement. Currently, your Funding Statement reads as follows:

“VCSFI 2024-25 funding was received for this study. “

Please include your amended statements within your cover letter; we will change the online submission form on your behalf.

5. Your ethics statement should only appear in the Methods section of your manuscript. If your ethics statement is written in any section besides the Methods, please move it to the Methods section and delete it from any other section. Please ensure that your ethics statement is included in your manuscript, as the ethics statement entered into the online submission form will not be published alongside your manuscript.

6. If the reviewer comments include a recommendation to cite specific previously published works, please review and evaluate these publications to determine whether they are relevant and should be cited. There is no requirement to cite these works unless the editor has indicated otherwise.

[Note: HTML markup is below. Please do not edit.]

Reviewers' comments:

Reviewer's Responses to Questions

**Comments to the Author**

1. Is the manuscript technically sound, and do the data support the conclusions?

Reviewer #1: Partly

Reviewer #2: Partly

**********

2. Has the statistical analysis been performed appropriately and rigorously?

Reviewer #1: I Don't Know

Reviewer #2: I Don't Know

**********

3. Have the authors made all data underlying the findings in their manuscript fully available?

The PLOS Data policy

Reviewer #1: No

Reviewer #2: Yes

**********

4. Is the manuscript presented in an intelligible fashion and written in standard English?

Reviewer #1: No

Reviewer #2: Yes

**********

Reviewer #1: The manuscript presents a relevant study, but several issues need clarification and improvement.

1. In bioanalytical HPLC assays, an internal standard is generally recommended to compensate for extraction variability, injection errors, and matrix effects. The authors should justify why no standard was used and discuss its possible impact on assay reliability and reproducibility.

2. Important pharmacokinetic parameters such as AUC, half-life, clearance, and volume of distribution were not evaluated. The authors should either expand the PK analysis or clearly state that this is a preliminary pilot study. The pharmacokinetic application is restricted only to Cmax and Cmin determination in six patients.

3. The clinical validation was conducted in only six breast cancer patients, which limits the statistical strength and generalizability of the findings. This limitation should be clearly acknowledged in the Discussion section.

4. The reported LLOQ (0.375 µg/mL) is higher than many previously reported LC-MS methods. The authors should better justify whether this sensitivity is sufficient for clinical pharmacokinetic and therapeutic monitoring applications.

5. Important chromatographic details such as retention time, column particle size, column temperature, total run time, and system suitability parameters were not reported. These details are necessary for the reproducibility of the method. Although matrix effect evaluation was mentioned, detailed quantitative data and acceptance criteria were not adequately presented. The authors should provide matrix factor calculations and variability data from different plasma lots.

6. Some freeze-thaw stability values exceeded 105-109%, which requires clarification. The authors should discuss the possible reasons for these variations and confirm compliance with FDA and ICH acceptance limits.

7. Important patient-related information, such as inclusion-exclusion criteria, concurrent medications, hepatic or renal status, and infusion duration, was not adequately described.

8. The authors are encouraged to provide raw data or validation datasets in a public repository or supplementary files.

Reviewer #2: The authors should add a high-resolution schematic illustration of the methodology and overall workflow to enhance the quality of the manuscript.

Additionally, they should also provide high-resolution versions of the graphs, figures, and chromatograms.

It is observed that the solvent used to prepare the paclitaxel stock solution has not been specified. Authors are required to mention the same.

The clinical application was conducted in only six patients, which may be insufficient to conclude. Validation for a higher number of patients is recommended.

The authors are recommended to provide the raw chromatography data files, along with the analyzed images/data outputs, as supplementary information.

A comparison of the proposed method with traditional methods should be presented in a tabular format, covering runtime, solvent consumption, extraction efficiency, sensitivity, and cost-effectiveness.

**********

what does this mean?). If published, this will include your full peer review and any attached files.

If you choose “no”, your identity will remain anonymous but your review may still be made public.

**Do you want your identity to be public for this peer review?**  For information about this choice, including consent withdrawal, please see our Privacy Policy

Reviewer #1: No

Reviewer #2: **Yes:**  Arun Saini

**********

[NOTE: If reviewer comments were submitted as an attachment file, they will be attached to this email and accessible via the submission site. Please log into your account, locate the manuscript record, and check for the action link "View Attachments". If this link does not appear, there are no attachment files.]

To ensure your figures meet our technical requirements, please review our figure guidelines: https://journals.plos.org/plosone/s/figures

You may also use PLOS’s free figure tool, NAAS, to help you prepare publication quality figures: https://journals.plos.org/plosone/s/figures#loc-tools-for-figure-preparation.

NAAS will assess whether your figures meet our technical requirements by comparing each figure against our figure specifications.

# Author comment: Author response to Decision Letter 1

**DOI:** 10.1371/journal.pone.0358891.r002

10 Jul 2026

I have uploaded the response to reviewers in tabular form.

Attachment Submitted filename: Response to Reviewers.doc

# Peer review: Decision Letter 1

**DOI:** 10.1371/journal.pone.0358891.r003

**Authors:** Rajesh Kumar Singh

7 Sep 2026

Development and clinical validation of HPLC method for precise quantification of paclitaxel in plasma from breast cancer patients

PONE-D-26-17670R1

Dear Dr. Rahim,

We’re pleased to inform you that your manuscript has been judged scientifically suitable for publication and will be formally accepted for publication once it meets all outstanding technical requirements.

Within one week, you’ll receive an e-mail detailing the required amendments. When these have been addressed, you’ll receive a formal acceptance letter and your manuscript will be scheduled for publication.

An invoice will be generated when your article is formally accepted. Please note, if your institution has a publishing partnership with PLOS and your article meets the relevant criteria, all or part of your publication costs will be covered. Please make sure your user information is up-to-date by logging into Editorial Manager at Editorial Manager® and clicking the ‘Update My Information' link at the top of the page. For questions related to billing, please contact billing support.

If your institution or institutions have a press office, please notify them about your upcoming paper to help maximize its impact. If they’ll be preparing press materials, please inform our press team as soon as possible -- no later than 48 hours after receiving the formal acceptance. Your manuscript will remain under strict press embargo until 2 pm Eastern Time on the date of publication. For more information, please contact onepress@plos.org.

Kind regards,

Rajesh Kumar Singh, Ph.D.

Academic Editor

PLOS One

Reviewers' comments:

Reviewer's Responses to Questions

**Comments to the Author**

Reviewer #1: (No Response)

Reviewer #2: All comments have been addressed

Reviewer #3: All comments have been addressed

**********

2. Is the manuscript technically sound, and do the data support the conclusions?

Reviewer #1: No

Reviewer #2: Yes

Reviewer #3: Yes

**********

3. Has the statistical analysis been performed appropriately and rigorously?

Reviewer #1: Yes

Reviewer #2: Yes

Reviewer #3: Yes

**********

4. Have the authors made all data underlying the findings in their manuscript fully available?

The PLOS Data policy

Reviewer #1: Yes

Reviewer #2: Yes

Reviewer #3: Yes

**********

5. Is the manuscript presented in an intelligible fashion and written in standard English?

Reviewer #1: Yes

Reviewer #2: Yes

Reviewer #3: Yes

**********

Reviewer #1: several points could further improve the scientific quality and reproducibility of the manuscript.

1. The quantitative matrix effect evaluation remains incomplete. Although the authors acknowledge this limitation, matrix factor calculations using multiple plasma lots, as recommended by current bioanalytical validation guidelines, have not been performed.

2. The comparison with previously published methods would be more informative in a tabular format. While the Discussion has been expanded, a concise comparison table summarizing runtime, sensitivity, solvent consumption, extraction procedure, recovery, and cost-effectiveness would improve readability.

3. Additional patient-related clinical information should be provided, particularly concurrent medications, infusion duration, and relevant hepatic/renal function parameters, to better interpret the clinical validation.

4. A schematic workflow of the experimental methodology would improve the presentation and help readers quickly understand the overall study design.

5. Please clarify whether incurred sample reanalysis (ISR) or repeat analysis of clinical samples was performed to further demonstrate assay reproducibility in real patient specimens.

6. The manuscript would benefit from a brief discussion on the applicability of the proposed HPLC method for routine therapeutic drug monitoring, including its advantages and limitations compared with LC-MS/MS in clinical laboratories.

7. The limitation of the small clinical sample size (n = 6) should be emphasized more clearly, and the conclusions should be interpreted cautiously as preliminary findings from a pilot study.

8. Consider discussing potential interference from paclitaxel metabolites or co-administered drugs, as these may influence assay specificity in clinical samples.

9.Some important chromatographic validation parameters (e.g., system suitability results) are available only in the supplementary material. Including a brief summary in the main manuscript would improve accessibility.

Reviewer #2: The manuscript is ready for final acceptance and publication, as all previous concerns and reviewer comments have been successfully addressed.

Reviewer #3: The Manuscript entitled" Development and clinical validation of HPLC method for precise quantification of paclitaxel in plasma from breast cancer patients" is interesting & significant for dose optimisation of paclitaxel for breast cancer patients. However some sentences are repeated in methodology & result section. Although, the authors have improvised the manuscript as per the suggestions of previous reviewers. The manuscript may be accepted with some improvement.

**********

what does this mean?). If published, this will include your full peer review and any attached files.

If you choose “no”, your identity will remain anonymous but your review may still be made public.

**Do you want your identity to be public for this peer review?**  For information about this choice, including consent withdrawal, please see our Privacy Policy

Reviewer #1: No

Reviewer #2: **Yes:**  Arun Saini

Reviewer #3: **Yes:**  Dr Vandana Yadav

**********

# Editor report: Acceptance letter

**DOI:** 10.1371/journal.pone.0358891.r004

**Authors:** Rajesh Kumar Singh

PONE-D-26-17670R1

PLOS One

Dear Dr. Rahim,

I'm pleased to inform you that your manuscript has been deemed suitable for publication in PLOS One. Congratulations! Your manuscript is now being handed over to our production team.

At this stage, our production department will prepare your paper for publication. This includes ensuring the following:

* All references, tables, and figures are properly cited

* All relevant supporting information is included in the manuscript submission,

* There are no issues that prevent the paper from being properly typeset

You will receive further instructions from the production team, including instructions on how to review your proof when it is ready. Please keep in mind that we are working through a large volume of accepted articles, so please give us a few days to review your paper and let you know the next and final steps.

Lastly, if your institution or institutions have a press office, please let them know about your upcoming paper now to help maximize its impact. If they'll be preparing press materials, please inform our press team within the next 48 hours. Your manuscript will remain under strict press embargo until 2 pm Eastern Time on the date of publication. For more information, please contact onepress@plos.org.

You will receive an invoice from PLOS for your publication fee after your manuscript has reached the completed accept phase. If you receive an email requesting payment before acceptance or for any other service, this may be a phishing scheme. Learn how to identify phishing emails and protect your accounts at https://explore.plos.org/phishing.

If we can help with anything else, please email us at customercare@plos.org.

Thank you for submitting your work to PLOS One and supporting open access.

Kind regards,

PLOS One Editorial Office Staff

on behalf of

Dr. Rajesh Kumar Singh

Academic Editor

PLOS One
